# QTG-Finder: A Machine-Learning Based Algorithm To Prioritize Causal Genes of Quantitative Trait Loci in Arabidopsis and Rice

**DOI:** 10.1534/g3.119.400319

**Published:** 2019-07-29

**Authors:** Fan Lin, Jue Fan, Seung Y. Rhee

**Affiliations:** Department of Plant Biology, Carnegie Institution for Science, Stanford, California 94305

**Keywords:** Arabidopsis, causal gene, machine learning, quantitative trait loci, rice

## Abstract

Linkage mapping is one of the most commonly used methods to identify genetic loci that determine a trait. However, the loci identified by linkage mapping may contain hundreds of candidate genes and require a time-consuming and labor-intensive fine mapping process to find the causal gene controlling the trait. With the availability of a rich assortment of genomic and functional genomic data, it is possible to develop a computational method to facilitate faster identification of causal genes. We developed QTG-Finder, a machine learning based algorithm to prioritize causal genes by ranking genes within a quantitative trait locus (QTL). Two predictive models were trained separately based on known causal genes in Arabidopsis and rice. An independent validation analysis showed that the models could recall about 64% of Arabidopsis and 79% of rice causal genes when the top 20% ranked genes were considered. The top 20% ranked genes can range from 10 to 100 genes, depending on the size of a QTL. The models can prioritize different types of traits though at different efficiency. We also identified several important features of causal genes including paralog copy number, being a transporter, being a transcription factor, and containing SNPs that cause premature stop codon. This work lays the foundation for systematically understanding characteristics of causal genes and establishes a pipeline to predict causal genes based on public data.

As the world’s population expands, food security faces a major challenge in the near future. By 2050, world population is projected to grow by 34%, which will require a 70% increase of global food production to meet the demand (The Food and Agriculture Organization 2009). To catch up with the growing global food demand, it is important to improve the efficiency of arable land usage by developing better crops.

Many agriculturally and medically important traits are quantitative and controlled by multiple genetic loci. Examples include plant height, grain yield, and flowering time in plants and common disorders such as cancer, diabetes, and hypertension in humans. The variation in quantitative traits allows organisms to adapt to various environments (Baxter *et al.* 2010; Leinonen *et al.* 2013). Quantitative traits are determined by a combination of genetic complexity and environmental factors (Mackay 2001). The genetic complexity of quantitative traits comes from the involvement of multiple quantitative trait loci (QTL) and the non-additive interactions among them (Carlborg and Haley 2004; Mackay 2014). Causal genes of QTL are genes whose differences in the DNA sequence or state cause a phenotypic variation in parental genotypes and are supported by multiple lines of experimental evidence including mutational analysis, transgenic complementation, and deficiency complementation (Weigel and Nordborg 2005). To understand the evolutionary forces and molecular mechanisms that shape the genetic architectures of adaptive traits, we need to identify all major causal genes that contribute to phenotypic variation of the traits and elucidate the molecular mechanisms of their actions. Achieving this goal will facilitate rational engineering of plant traits and more accurate prediction of the effects of their modifications on the engineered plant.

QTL linkage mapping and genome wide association study (GWAS) are two common approaches used to identify QTL, each with its own strengths and limitations. Both mapping approaches are based on the co-segregation of a trait and genetic variants in a population. The population for linkage mapping is usually the progeny of parental plants that differ in a trait, such as an F2 population or recombinant inbred lines (Bergelson and Roux 2010). GWAS mapping uses a natural population that has a heritable variation of a trait. Compared to GWAS, linkage mapping does not suffer from issues like population structure and suffers less from rare alleles (Bergelson and Roux 2010). For example, the most significant seed dormancy QTL *DOG1* identified by linkage mapping was not identified by GWAS, likely due to the rarity of the strong allele in the GWAS population (Bentsink *et al.* 2010; He 2014). Confounding population structure can cause a high false positive rate in GWAS, though some methods have been developed to ameliorate it (Price *et al.* 2010). However, efforts to correct it could result in a higher false negative rate (Brachi *et al.* 2010). Linkage mapping is less prone to these issues, but it cannot identify QTL of minor effects when the sample size is small (Otto and Jones 2000; Xu 2003; Martin and Orgogozo 2013; Wellenreuther and Hansson 2016).

For QTL identified by linkage mapping, finding causal genes underlying them is still a big bottleneck (Bergelson and Roux 2010). In a typical rice linkage mapping, the size of a QTL can range from 200kb- 3Mb, which can harbor tens to hundreds of genes depending on the mapping population and gene density (Bargsten *et al.* 2014; Daware *et al.* 2017). Even in the post-genomic era where all the genes in the genome are uncovered, identifying QTL causal genes is not straightforward since many QTL either contain no obvious candidate genes or too many genes potentially relevant for the trait (Nuzhdin *et al.* 1999). Therefore, despite the many QTL that have been reported in plants, only a few have been studied at the molecular level.

To narrow down the range of candidate genes in a QTL region, conventional fine mapping is reliable but time-consuming and labor-intensive. The basis of fine mapping is to create a population that has more recombination events within a QTL in order to identify a smaller genomic segment that co-segregates with the trait. However, the enormous time and labor required for creating and screening a population of progenies limits the usage of this method (Tuinstra *et al.* 1997). Depending on the frequency of recombination, thousands of progenies may need to be genotyped to get to a gene-scale resolution (Dinka *et al.* 2007). For example, 1,160 progenies were screened to identify the *Pi36* gene in rice and as many as 18,994 progenies were screened to identify the causal gene of *Bph15* in rice (Yang *et al.* 2004; Liu *et al.* 2005). The high cost associated with genotyping and phenotyping makes it challenging to apply fine mapping to all QTL.

Alternative approaches to refine the candidate list of causal genes include meta-analysis, joint linkage-association analysis, and other computational methods including machine-learning algorithms. The first two approaches require either the availability of many QTL studies on similar traits or an additional association mapping experiment (Buckler *et al.* 2009; Motte *et al.* 2014; Yin *et al.* 2017). Computational methods including machine-learning algorithms have been developed to prioritize disease associated genes and genetic variants in human (Perez-Iratxeta *et al.* 2002; Kircher *et al.* 2014; Ritchie *et al.* 2014; Hormozdiari *et al.* 2015). To distinguish disease-associated from non-associated variants, a variety of information has been used, including the effect of polymorphism (Ng and Henikoff 2003; Kircher *et al.* 2014; Gelfman *et al.* 2017), sequence conservation (Pollard *et al.* 2010; Huang *et al.* 2017), regulatory information (Deo *et al.* 2014), expression profile (Mordelet and Vert 2011; Deo *et al.* 2014), Gene Ontology (GO) (Mordelet and Vert 2011), KEGG pathway (Mordelet and Vert 2011), and publications (Perez-Iratxeta *et al.* 2002). In contrast, only two causal gene prioritization approaches are available for plants. One method was developed for GWAS in maize based on co-expression networks (Schaefer *et al.* 2018). Another method was developed for linkage mapping based on biological process GOs (Bargsten *et al.* 2014). To date, no machine-learning approach using multiple data types has been developed to address this problem.

Here, we built a supervised learning algorithm to prioritize QTL causal genes using known causal genes in *Arabidopsis thaliana* (Arabidopsis) and *Oryza sativa* (rice) and a suite of publicly available genetic and genomic data. For each species, we trained a predictive model using features based on polymorphism data, function annotation, co-function network, and paralog copy number. By testing the models on an independent set of known causal genes, we demonstrated its efficacy in prioritizing causal genes.

## Materials and Methods

### Data sources and features used in QTG-Finder

Twenty-eight features were extracted from published genome-scale data, which included eight polymorphism features, seventeen functional annotation features, one co-function network feature and two evolutionary features (Supplementary Table S3).

Arabidopsis polymorphism data of 1,135 accessions was downloaded from 1001 Genomes Project (https://1001genomes.org) (1001 Genomes Consortium 2016) and rice polymorphism data of 3,010 cultivars was downloaded from Rice SNP-Seek Database (http://snp-seek.irri.org) (Mansueto *et al.* 2017). We used SIFT4G (v 2.4) (Ng and Henikoff 2003) and SnpEff (v 4.3r) (Cingolani *et al.* 2012) to annotate the raw polymorphism data. The number of non-synonymous SNP as annotated by SIFT4G was normalized to protein length and used as a numeric feature (normalized_nonsyn_SNP). Non-synonymous SNPs at conserved protein sequences were predicted to cause deleterious amino acid changes by SIFT4G. The presence of deleterious non-synonymous SNPs in a gene was used as a binary feature (is_nonsyn_deleterious). If a gene contained any deleterious non-synonymous SNPs, the “is_nonsyn_deleterious” feature was set to 1, otherwise it was set to 0. Other binary polymorphism features such as “is_start_lost” (start codon lost) and “is_start_gained” (start codon gained) were extracted from SnpEff annotations in the same way. For “is_SNP_cis”, the Position Weight Matrices of *cis*-elements were downloaded from CIS-BP database (Build 1.02) (Weirauch *et al.* 2014) and mapped to 1kb upstream of all genes in the genome using FIMO (v 4.12.0) (Grant *et al.* 2011). The *cis*-elements with a matching score above 55 were imported into SnpEff library to annotate the SNPs. This matching score cutoff was determined by a cross-validation as described later.

Functional annotation features were binary features based on GO (Gotz *et al.* 2008; Jones *et al.* 2014) and Plant Metabolic Network (PMN) (Schläpfer *et al.* 2017). Arabidopsis and rice genes were annotated by Blast2GO (BLAST+ 2.2.29) and InterProScan (v 5.3-46.0). The molecular function GOs were then converted to high-level functional groups such as transcription factor, receptor, kinase, transporter, and enzyme to mitigate the effect of some inaccurate annotations (Jones *et al.* 2007). To assess the performance of this approach, we compared aggregated high-level GO annotations from Blast2GO with aggregated high-level curated GO annotations from AmiGO (http://current.geneontology.org/products/pages/downloads.html) and un-aggregated GO annotations. Genes annotated as enzymes were further classified into 13 PMN metabolic domains such as carbohydrate metabolism and nucleotide metabolism (Schläpfer *et al.* 2017). Unclassified genes in PMN were classified as “is_other_metabolism”. Genes annotated as enzymes by GO but not present in PMN databases are either enzymes involved in macromolecule metabolic processes or enzymes without a specific function assigned. Since the majority of them are involved in macromolecule metabolic processes, we named this group as “is_macromolecule_metabolism”.

Co-function networks of Arabidopsis and rice were retrieved from AraNet and RiceNet (Lee *et al.* 2010; Lee *et al.* 2011). The sum of all the edge weights of a gene was used as the “network_weight” feature. We used the sum of edge weights because hub genes have been proposed to be hotspots of phenotypic variation (Martin and Orgogozo 2013).

Paralog copy number (paralog_copy_number) and essential gene prediction (is_essential_gene) were taken from a previous publication (Lloyd *et al.* 2015).

### Arabidopsis and rice causal genes used for training and independent validation

For model training and cross-validation, curated causal genes from Martin and Orgogozo were used as positives for algorithm training (Martin and Orgogozo 2013). In total, 60 Arabidopsis and 45 rice causal genes were used as the initial training set (Supplementary Tables S1 and S2). We curated and included gene identifiers and trait categories in these tables (Supplementary Methods). For literature validation, we performed a further literature curation and found eleven Arabidopsis and eighteen rice causal genes, which were not included in the Martin and Orgogozo list (Supplementary Methods and Supplementary Table S8).

The QTL regions used for independent validation were obtained from previously published studies. Even though some studies fine mapped the QTL, we still used the original QTL regions instead of the fine-mapped regions since our method was developed to replace fine mapping. We included all genes between the markers that were used to define a QTL for prioritization. When the genome locations of the markers were not provided in the publication, we searched their genome locations in Gramene marker database (https://archive.gramene.org/db/markers/marker_view).

### Algorithm training and parameter optimization

**Figure 1:**
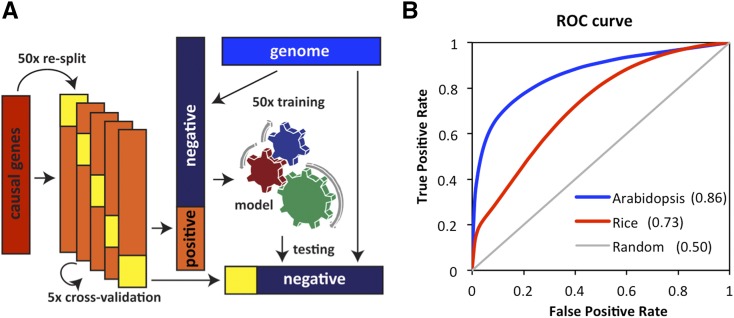
Model training and optimization based on cross-validation. (A) model training and cross-validation framework. We randomly selected negatives from the genome and iterated to maximize the combinations of training and testing data. (B) The ROC curve of Arabidopsis and rice models after parameter optimization. True and false positive rates were based on the average of all iterations. The gray diagonal line indicates the expected performance based on random guessing. The number in parentheses indicates Area Under the ROC Curve (AUC-ROC).

The QTG-Finder algorithm was developed in Python (v 3.6) with the ‘sklearn’ package (v 0.19.0) (Pedregosa *et al.* 2011). We developed an extended 5-fold cross-validation framework (Figure 1A) to evaluate training performance and optimize model parameters.

For the 5-fold cross validation, curated causal genes were used as positives and the other genes from the genome were used as negatives. The positives were randomly re-split into training and testing positives in a 4:1 ratio and in an iterative manner. Training and testing positives were combined with different sets of negative genes that were randomly selected from the rest of the genome. To increase the combination of positives and negatives, we re-split the positives 50 times randomly and selected negatives 50 times. This number of iterations ensured greater than 99% probability that every positive sample co-occurred with every negative at least once in the training or testing set during the cross-validation process. The probability of co-occurrence was calculated as Equation 1. *P_co_* is the probability of co-occurrence of a positive and a negative in a testing or training set. *N* is the total number of negative samples. *n* is the number of negative samples selected as testing or training samples. *R* is the number of iterations used to re-split the positive set. *C* is the number of cross-validation folds that contains a positive sample. *C* was set to 4 for the training set and 1 for the testing test. *S* is the number of iterations to randomly select the negative set.

$$P_{co}=1-{\left[\prod_{i=0}^{n}\left(1-\frac{1}{N-i}\right)\right]}^{R*C*S}$$ (1)

We tested different classifiers and parameters and optimized the model based on Area Under the Curve of the Receiver Operating Characteristic (AUC-ROC). The average AUC-ROC from all iterations was used to evaluate training performance. We tested three classifiers: Random Forest, naïve Bayes, and Support Vector Machine (Cortes and Vapnik 1995; Tin Kam 1998; Zhang 2004) (Supplementary Figure S1). For Random Forest, we tuned the number of trees and the maximum number of features for each tree based on AUC-ROC (Supplementary Figure S2). We used 100 trees and a max_feature of 9 for Random Forest. For Support Vector Machine, the RBF kernel was used and the C parameter was tuned. Random Forest was chosen for further analysis since its performance was slightly better than the other two classifiers. The ratio of positives and negatives in training data were also tuned to maximize cross-validation AUC-ROC (Supplementary Figure S3). The best performing positives:negatives ratio was 1:20 for Arabidopsis and 1:5 for rice. For testing, a positives:negatives ratio of 1:200 was used since it is close to the average ratio of causal and non-causal genes in real QTL.

### Feature importance analysis

We implemented a leave-one-out analysis to evaluate feature importance. This method was based on the change of AUC-ROC (ΔAUC-ROC) when leaving out one feature from the models. The same cross-validation framework was used for this analysis. For each iteration, we calculated AUC-ROC on the original and the leave-one-out models developed with the same training and testing datasets. The ΔAUC-ROC was calculated by subtracting the leave-one-out AUC-ROC from the original AUC-ROC. With the results from all iterations, we calculated the average ΔAUC-ROC for each feature.

### Independent literature validation

For validation, we applied the models to an independent set of causal genes that were curated from recent literature and not used for cross-validation. The models were trained by all known causal genes from the initial list and negatives were randomly selected from the rest of the genome. Model training was repeated 5,000 times using resampled training negatives from the genome in combination with the same set of known causal genes. The 5,000 iterations were conducted to ensure that there was >99% probability that each gene in the genome was selected at least once. We applied the models to each of the independent causal gene and all other genes located within the QTL. All genes within the QTL were ranked based on the frequency of being predicted as a causal gene.

### Model performance for multiple QTL

To understand performance of the model when it was applied to multiple QTL of the same trait, we conducted simulations. We calculated the probability of including at least *K* causal genes within the prioritized list at a given cut-off of the rank percentile when applying the models to a total of *N* QTL with Equation 2. *p* is the probability of a known causal gene to be included at a particular cutoff of the prioritized list using the independent set of causal genes found in the literature. *x* is the number of causal genes included in the cut-off.

$$P\left(x\geq K\right)=\sum_{x=K}^{N}\left(\begin{array}{c} N\\ x \end{array}\right)p^{x}{\left(1-p\right)}^{N-x}$$ (2)

### Trait category analysis

The trait category analysis was performed in a similar way as the independent literature validation except using different training and testing sets. Each curated causal gene was tested once. For each round, one curated causal gene was removed from the training set. Then the model was trained and applied to rank the removed causal gene and 200 flanking genes.

### Data Availability

The source code for QTG-Finder and related analyses such as cross-validation, feature importance analysis, and trait category analysis are available at https://github.com/carnegie/QTG_Finder. Supplemental material available at FigShare: https://doi.org/10.25387/g3.8968769.

## Results

### QTG-Finder: a machine-learning algorithm to prioritize causal genes

We developed the QTG-Finder algorithm to accelerate finding causal genes from QTL data and generated two predictive models in Arabidopsis and rice. These two species were selected for model training since they have the largest number of QTL causal genes (QTGs) that have been discovered by fine mapping and map-based cloning in plants (Martin and Orgogozo 2013). For model training, we selected 60 Arabidopsis and 45 rice causal genes as a positive set (Martin and Orgogozo, 2013, Supplementary Tables S1 and S2). The negative set was a set of genes randomly selected from the rest of the genome. To train the models, we used 28 Arabidopsis features and 27 rice features, including polymorphism features, functional categories of genes, function inference from co-function networks, gene essentiality, and paralog copy number (Supplementary Tables S3, S4 and S5). These features were generally independent from each other; most have a Pearson’s correlation coefficient <0.2 (Supplementary Figure S4).

We devised an extended cross-validation framework to optimize the models (Figure 1A). With this framework, we evaluated the training performance with AUC-ROC and optimized parameters. We used AUC-ROC for model optimization since our goal is not only to identify causal genes (true positives) in the prioritized list but also reduce the number of candidates by eliminating non-causal genes (true negatives) from the prioritized list. To find the optimal parameters, we compared the AUC-ROC of different machine-learning classifiers, modeling parameters, the ratio of positive:negative genes in the training set, and different methods to generate GO features (Supplementary Figures S2, S3, S4, and S5). Random Forest was selected as the classifier since it was less prone to over-fitting and performed better than the other classifiers tested (Supplementary Figure S1). After optimization, AUC-ROC for the Arabidopsis and rice models were 0.86 and 0.73, respectively (Figure 1B). The optimized models were also evaluated by confusion matrix (Supplementary Table S6). The true positive and true negative rates calculated from the confusion matrix indicated that the model was better at classifying non-causal genes than causal genes.

Since the positive training set used was relatively small, we also evaluated the relationship between training performance and size of the training set. The AUC-ROC increased as a larger training set was used. Interestingly, maximum gain in the AUC-ROC was achieved with 20 causal genes for the traits represented by the training set (Supplementary Figure S6).

### Important features for predicting causal genes

With the optimized models, we asked which features were important for causal gene prediction. Since Random Forest uses features and their interactions for classification (Touw *et al.* 2013), the importance of a feature cannot be measured by simple enrichment or depletion of a single feature in causal genes. Therefore, we evaluated feature importance based on the change of AUC-ROC (**Δ**AUC-ROC) when excluding a feature from the model (Lloyd *et al.* 2015). When an important feature is excluded from the model, the AUC-ROC should decrease.

**Figure 2:**
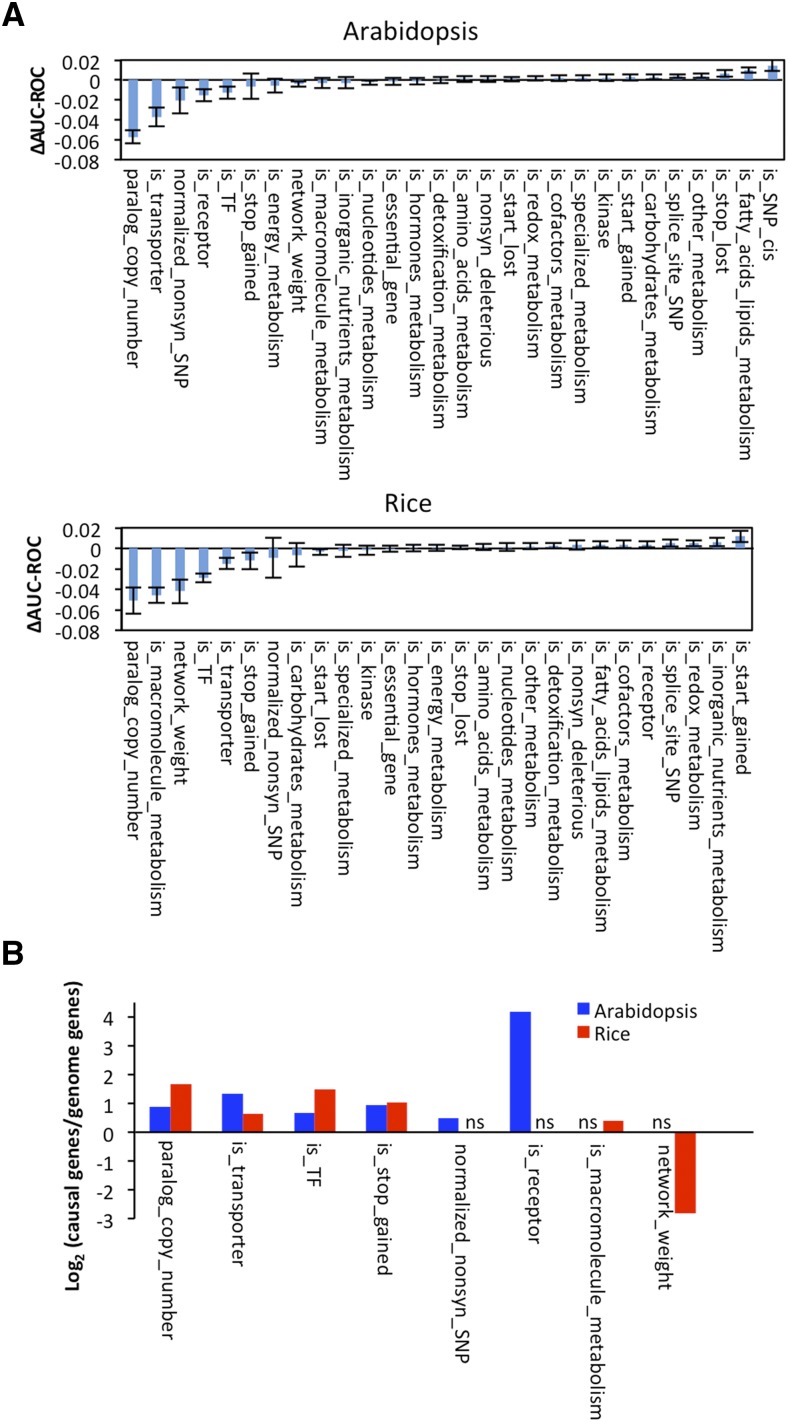
Important features of causal genes and their enrichment or depletion relative to the genome background. (A) Feature importance as indicated by the change of AUC-ROC (ΔAUC-ROC) when excluding each feature. The ΔAUC-ROC indicates the average value of all iterations. Error bars indicate standard deviation. The features with a name that starts with “is_” are binary variables. (B) The enrichment or depletion of the top 6 features in Arabidopsis and rice models. The enrichment/depletion were indicated by the ratio of causal genes to genome background. ns, not shown because the feature is not one of the top 6 features in that species.

For both Arabidopsis and rice models, eight features decreased AUC-ROC when removed (Figure 2A and Supplementary Table S7). The six most important features for Arabidopsis were paralog copy number, transporter, the number of non-synonymous SNPs normalized to protein length (normalized_nonsyn_SNP), receptor, transcription factor, and SNPs causing premature stop codon (is_stop_gained) (Figure 2A). The six most important features for rice were paralog copy number, macromolecule metabolism, network weight sum, transcription factor, transporter, and SNPs causing premature stop codon (is_stop_gained). Four out of the six most important features were consistent between Arabidopsis and rice models, which were paralog copy number, transporter, transcription factor, and SNPs causing premature stop codon.

For the six most important features in Arabidopsis and rice, we examined their ratio in known causal genes *vs.* randomly selected genes in the genome (Figure 2B). Compared to other genes in the genome, the causal genes in both species tended to have more paralogs, higher frequency of being a transporter or a transcription factor, and higher frequency of containing SNPs that cause premature stop codons. In addition, Arabidopsis causal genes were more likely to be a receptor and rice causal genes were more likely to be a non-hub gene.

The rest of the features contributed less to, but did not impair much, the model performance (**Δ**AUC-ROC< 0.02). Since there was no strong evidence that they impair prediction, we did not remove them from the models for further analysis.

### Validating QTG-Finder by ranking an independent set of QTL genes

To assess the predictability of QTG-Finder models, we searched the literature for a separate set of known causal genes from the initial training set. We found eleven Arabidopsis and eighteen rice genes that are likely causal genes underlying QTL when interpreting linkage mapping with additional evidence such as functional complementation, fine mapping, joint linkage-association analysis or genetic analyses (Supplementary Table S8). These causal genes were not used for model training or cross-validation.

To examine model performance independently, we applied the QTG-Finder models to this new set of causal genes. For each known causal gene, we ranked all the genes within its QTL region, based on the frequency of being predicted as a causal gene from 5,000 iterations. Since the number of genes in a QTL region varies, we used a gene’s rank percentile for evaluation. The rank percentile of a gene indicates the percentage of QTL genes that had higher ranks than the gene of interest.

**Figure 3:**
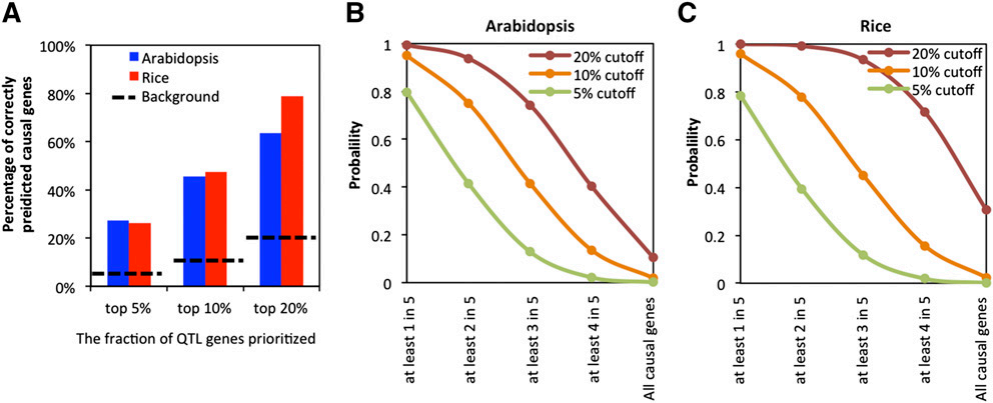
Model performance at different thresholds. (A) Percentage of recalled causal genes of a single QTL at different rank thresholds. Dashed lines indicate the background of random selections. (B-C) The probability of causal gene recall when analyzing multiple QTL simultaneously.

Based on the rank percentile of these known causal genes, we evaluated model performance at different cutoffs of rank percentile such as 5%, 10%, or 20%. We calculated the percentage of known causal genes being recalled at different cutoffs (Figure 3A). The top 20% of the ranked genes included seven Arabidopsis (∼64%) and fourteen rice (∼79%) causal genes (Supplementary Table S8). This set included 10-100 non-causal genes. With a more stringent cutoff of 5%, four Arabidopsis (∼27%) and three rice (∼26%) causal genes were prioritized. We examined the molecule types, trait categories, and features of the eight known causal genes (4 Arabidopsis and 4 rice) that were not prioritized within the top 20%, but did not observe any special trend.

We also asked whether the different strengths of experimental evidence of the causal genes affected the model performance. Causal genes were grouped based on the type of supporting experimental evidence (Table S6). The first group included genes with weak supporting evidence such as mutational analysis. The second group contained genes supported by stronger evidence such as fine mapping, functional complementation, and joint linkage-associate analysis. The average rank percentile of the two groups was similar; 18% for the first group and 16% for the second.

Since most linkage mapping studies identify multiple QTL, we asked what the probability is of identifying causal genes from multiple QTL simultaneously. We calculated a theoretical model performance on multiple QTL identification as the probability of identifying causal genes for at least *N* QTL when applying the model to all QTL of a trait (Figure 3B and C). For example, assuming there were five QTL (*N* = 5) of a trait identified by a linkage mapping study and each QTL contained one causal gene. For the Arabidopsis model, the probability of identifying at least one causal gene would be 99% when the top 20% genes of all QTL were tested experimentally. The probability of identifying all five causal genes would be 10% when the top 20% cutoff was used. We further compared the performance of all three cutoffs, top 20%, top 10%, and top 5%. The probability of identifying at least one out of five causal genes would be no less than 80% for all three cutoffs. However, the probability to recall at least four out of five causal genes at top 20% would be 40%, 14% (at top 10%), and 2% (at top 5%). Therefore, a less stringent cutoff, top 20%, performs much better than a more stringent cutoff if one is interested in finding most of the causal genes or causal genes of a particular QTL. However, if the goal is to identify any causal gene, then screening the top 5% of all QTL may be a more strategic approach.

To compare our results with an existing QTL prioritization method for rice (Bargsten *et al.* 2014), we examined how genes in our rice validation set were prioritized in that study. Only three out of eighteen genes were prioritized as candidates when the top 9% genes in QTL regions were considered. For QTG-Finder, eight out eighteen genes were prioritized as candidates when the top 9% genes were considered.

### Trait type preference of QTG-Finder models

**Figure 4:**
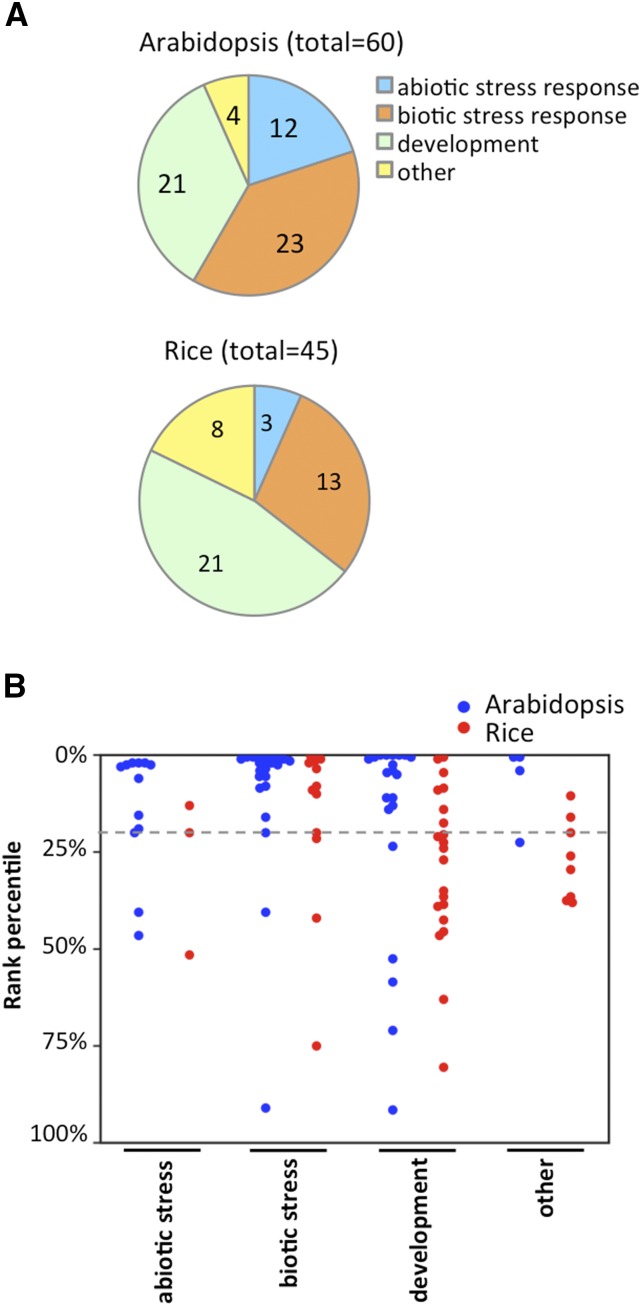
Performance comparison across trait categories. (A) Trait categories of known causal genes from the training set. (B) The rank percentile of causal genes of different trait categories. Each causal gene and 200 neighboring genes were used as testing set only once. All other known causal genes were used for training. Each dot indicates a known causal gene. The gray dashed line indicates 20% rank percentile. The trait categories of causal genes are defined in Tables S1 and S2.

Since the training set included genes for different types of traits at an imbalanced ratio, we asked how QTG-Finder models would work for each type of traits (Figure 4A). The independent validation in the previous section was based on causal genes related to plant development and disease resistance (Supplementary Table S8). However, this validation set was not large enough for a systematic analysis and did not have any abiotic-stress-related causal genes. Therefore, we performed a rank analysis for different trait categories using the known causal genes from the initial training set (60 for Arabidopsis and 45 for rice). For this rank analysis, each causal gene was removed from the training set once and used for a rank test. The removed causal gene and its 200 neighboring genes in the genome were used as a testing set. We applied the models to each testing set to obtain the rank for each causal gene. Then we calculated the average rank for the causal genes in the four trait categories: development, abiotic stress, biotic stress and “other”. The “other” category included traits in seed hull color, oil composition, necrosis, etc. (Supplementary Tables S1 and S2).

Model performance varied for different trait categories. Both abiotic and biotic stress traits had better performance than developmental traits (Figure 4B). This could be because the developmental trait category has more diversified traits and genes than the abiotic and biotic stress trait category. In addition, the Arabidopsis model performed slightly better than the rice model for all trait categories. This trait category analysis can guide us to determine rank cutoffs when applying models to different types of traits.

## Discussion

Linkage mapping is a useful tool to identify the genomic regions responsible for many agriculturally and medically important traits. However, it is not straightforward to identify the genes that cause phenotypic variation of the trait from these genomic regions. The discovery of causal genes still requires time-consuming and labor-intensive fine mapping. In this study, we developed a machine-learning algorithm to reduce the number of candidates to be tested experimentally in order to accelerate the discovery of causal genes.

### A machine-learning algorithm to prioritize QTL causal genes

Several causal variant or gene prioritization methods have been developed for human data but not many in plants (Bargsten *et al.* 2014; Kircher *et al.* 2014; Jagadeesh *et al.* 2016; Schaefer *et al.* 2018). Most prioritization methods have been developed for GWAS mapping in human, an organism where linkage mapping cannot be performed. However, linkage mapping can capture rare alleles and has been broadly used to study quantitative traits of livestock, crops, and model organisms. A causal gene prioritization is especially helpful for large QTL identified by linkage mapping, which can constitute tens to thousands of genes. One method has been developed in rice to prioritize causal genes for linkage mapping (Bargsten *et al.* 2014). This method is based on the hypothesis that causal genes from multiple QTL of the same trait are more likely to have the same biological process GO terms, and therefore genes with overrepresented biological process GOs were prioritized as causal genes. However, this method gives no predictions for ∼15% of traits and lack an unbiased performance evaluation since the same set of causal genes was used to determine the cutoff and evaluate performance. We evaluated performance of this GO-based method with the causal genes from the validation set used in this study. The GO-based method identified fewer causal genes compared to QTG-Finder when a similar fraction of QTL was prioritized. Another method named Camoco has been developed in maize to prioritize causal genes for GWAS (Schaefer *et al.* 2018). Camoco prioritizes genes based on the relative strength and degree of co-expression among genes near GWAS peaks. The success of this method depends highly on the gene expression dataset being appropriate for the trait of interest. For example, an expression dataset of root tissues may work better for root-related traits than shoot-related traits. In addition, this method may not be able to capture causal genes that are transiently expressed or expressed at low levels (Moyers 2018). Since each of these approaches utilizes different sets of information, they may be used in conjunction with the QTG-Finder.

In this study, we built a supervised learning algorithm using multiple features and validated its efficacy with an independent dataset from the literature. The models could accelerate the discovery of causal genes by ranking all the genes in a QTL region and prioritizing the top 5%, 10%, or 20% genes, which are most likely to contain the causal gene, for experimental testing. Based on an assessment using independent data in the literature, we calculated the performance when applying the models to all QTL of a trait and compared three cutoffs (top 5%, 10%, and 20%). The less stringent cutoff (top 20%) had a higher chance to find more causal genes (Figure 3B and C) but yielded more candidates that needed to be tested by experiments. The more stringent cutoff (top 5%) had a lower chance to find all causal genes but yielded a smaller set of candidates to test. The probability for the models to find at least one causal gene is high for all three cutoffs. If the goal were to find at least one causal gene for functional studies and the particular QTL regions did not matter, the 5% cutoff would be more efficient. If the goal were to discover all causal genes and understand the genetic architecture of a trait, the 20% cutoff would be better. Similarly, if a particular QTL were of interest for discovering the underlying causal gene, the 20% cutoff would be better.

There are several conceptual and practical advantages of QTG-Finder algorithm. First, this algorithm combines multiple types of publically available data including polymorphisms, function annotations, co-function network and other genomic data, which have not been applied to prioritize causal genes from linkage mapping studies. Second, models were trained on causal genes from various traits and can be applied to several types of traditional traits, though the prioritization efficiency was not equivalent. Third, validation from the literature provides guidance on what proportion of genes to prioritize in practice rather than arbitrarily selecting a threshold. Fourth, the models treat each QTL independently and have the flexibility to prioritize a specific QTL of interest.

Two limitations of this study are the small number of known causal genes in plants and the impurity of negative set used for model training. As a positive dataset, we used 60 Arabidopsis and 45 rice causal genes that have been verified by map-based-cloning. Even though the positive dataset are of high quality, the sample size may not be large enough to represent all categories or features of causal genes and therefore lead to ascertainment bias. The models may perform better on over-represented gene categories or features in the training set. A larger positive training set will mitigate this bias. For example, the qTARO database is a useful resource to find potential new causal genes for rice, though these genes would need to be curated further (Yonemaru *et al.* 2010). The negative set was composed of genes randomly selected from the rest of the genome. Though we excluded known causal genes, there could still be some uncharacterized causal genes. As a result of these limitations, 20% cutoff will still yield ∼100 candidates for large QTL, which is challenging for experimental characterization unless at least a medium-throughput phenotyping method is available. Fortunately, plant science is entering an era of high-throughput phenotyping with advances in automation, computation and sensor technology (Fahlgren *et al.* 2015; Araus *et al.* 2018). Our study establishes an extendable framework that can be easily updated with new training datasets and features. As more causal genes are uncovered, the new data can be easily incorporated to improve the models.

The current models included genes in the reference genomes of rice and Arabidopsis. Even though the majority of causal genes are present in the reference genomes, there are exceptions. For example, *SUB1A*, *SNORKEL1*, and *SNORKEL2* are causal genes absent in the rice reference genome Nipponbare (Xu *et al.* 2006; Hattori *et al.* 2009). Those genes cannot be predicted with the current models. In the future, this could be addressed by using pan-genome gene information and presence–absence variation (Zhao *et al.* 2018).

### Important features for predicting QTL causal genes

Many causal genes were repeatedly found to cause phenotypic variation of similar traits, which is also known as genetic hotspots of phenotypic variation or gene reuse (Martin and Orgogozo 2013). By examining 1,008 causal alleles in animals, plants, and yeasts, Martin and Orgogozo found *de novo* mutations to occur repeatedly at certain genes or orthologous loci and causing similar phenotypic variations either among lineages or within a single lineage. The prevalence of gene reuse suggests that causal genes are likely to have some genetic and genomic characteristics that allow them to be repeatedly used for phenotypic variation. The mechanism for gene reuse is not clear but it may be influenced by factors such as the availability of standing genetic variation, mutation rate, pleiotropic constraint, and epistatic interactions of a gene (Conte *et al.* 2012; Conte *et al.* 2015).

While many QTL causal genes have been cloned, their features have not been systematically examined before. Instead of evaluating each feature individually, we trained Random Forest models and evaluated feature importance for all features by adopting the leave-one-out strategy. Several of the most important features were consistent between Arabidopsis and rice models: containing SNPs that cause a premature stop codon, paralog copy number, being a transporter, and being a transcription factor.

We extracted polymorphism features from re-sequencing data of many accessions, which provide more information about the existence of standing genetic variation in the species than the polymorphism data used for linkage mapping, which typically comes from two parental lines. DNA polymorphisms such as nonsense SNPs, deleterious non-synonymous SNPs, SNPs at *cis*-regulatory elements, and SNPs at splice junctions have been used as features to classify causal and non-causal variants of human diseases (Kircher *et al.* 2014; Jagadeesh *et al.* 2016). These SNPs can directly affect the function or expression of a gene and therefore are more likely to be causal than the rest of the SNPs. Our results indicate Arabidopsis and rice causal genes were more likely to carry a SNP that causes premature stop codon (nonsense SNP) than an average gene in the genome. We also found Arabidopsis causal genes were more likely to have more non-synonymous SNPs than an average gene in the genome. Besides the high impact SNPs in coding regions, we also examined polymorphisms in non-coding regions since about 90% of human trait/disease-associated SNPs are located outside of coding regions (Hindorff *et al.* 2009). The SNPs at *cis*-regulatory elements did not show a high feature importance in our algorithm, although this might be due to limited exploration of non-coding sequences in plants. The CIS-BP database contains *cis*-elements of 44% of the transcription factors in Arabidopsis (Weirauch *et al.* 2014). Developing a more accurate and complete map of functional non-coding regions based on conserved noncoding sequences (Van de Velde *et al.* 2014) will potentially make non-coding polymorphism features more useful for prioritizing causal genes in the future. The SNPs linked to causal SNPs might add background noise and reduce the capability to distinguish causal genes from non-causal genes. This could be a reason why half of the polymorphism features were not significantly enriched in the causal genes (Supplemental Table S3).

Paralogs contribute to the evolution of plant traits by providing functional divergence that gives plants the potential to adapt to complex environments (Panchy *et al.* 2016). Through evolution, genes involved in signal transduction and stress response have retained more paralogs while essential genes like DNA gyrase A have retained fewer paralogs (Lloyd *et al.* 2015; Panchy *et al.* 2016). By acquiring new functions or sub-functions, paralogs allow plants to sense and handle different environmental conditions in a more comprehensive and adjustable way. For example, the eight paralogous heavy metal ATPases (HMAs) in Arabidopsis are all involved in heavy metal transport but have different substrate preferences, tissue expression patterns, and subcellular compartment locations (Takahashi *et al.* 2012). Three of them, *HMA3*, *HMA4*, *HMA5*, are known causal genes of QTL identified by linkage mapping. The known causal genes we analyzed have more paralog copies than other genes in the genome. This suggests that many plant causal genes are playing a role in providing more phenotypic tuning parameters to allow plants to adapt to complex environments.

When the training set is small, there is a possibility of ascertainment bias. For example, the GO features, is_transporter and is_transcription_factor, may be considered as important features because of their enrichment in the current training set. We will have more confidence of the importance of features when more known causal genes become available.

The important features of causal genes identified by linkage mapping may be different from those identified by GWAS. Given the difference of the two genetic approaches, linkage mapping tends to identify large-effect alleles of protein-coding regions, while GWAS tends to identify common alleles with a wider range of effect sizes at protein-coding regions or non-coding regions (Singleton *et al.* 2010). Therefore, whether the features used in this study can be applied to GWAS remains open. It would be interesting to systematically compare causal genes identified by linkage mapping and GWAS in the future.

Overall, QTG-Finder is a novel machine-learning pipeline to prioritize causal genes for QTL identified by linkage mapping. We trained QTG-Finder models for Arabidopsis and rice based on known causal genes from each species, respectively. By utilizing information like polymorphisms, function annotations, co-function networks, and paralog copy numbers, the models can rank QTL genes to prioritize causal genes. Our independent literature validation demonstrates that the models can recall about 64% of causal genes for Arabidopsis and 79% for rice when the top 20% of ranked QTL genes were considered. The algorithm is applicable to any traditional quantitative traits but the performance was different for each trait type. Since QTG-Finder is a machine-learning based pipeline, extending the training set and adding features can easily expand and improve the models. We envision that frameworks like QTG-Finder can accelerate the discovery of novel quantitative trait genes by reducing the number of candidate genes and efforts of experimental testing.
